# The Keratinocyte as a Crucial Cell in the Predisposition, Onset, Progression, Therapy and Study of the Atopic Dermatitis

**DOI:** 10.3390/ijms221910661

**Published:** 2021-10-01

**Authors:** Pamela Gallegos-Alcalá, Mariela Jiménez, Daniel Cervantes-García, Eva Salinas

**Affiliations:** 1 Department of Microbiology, Center of Basic Science, Autonomous University of Aguascalientes, Aguascalientes 20100, Mexico; pamela.g.alcala@gmail.com (P.G.-A.); mayojv@hotmail.com (M.J.); cervantes.daniel@gmail.com (D.C.-G.); 2 National Council of Science and Technology, Ciudad de México 03940, Mexico

**Keywords:** keratinocyte, atopic dermatitis, allergic inflammatory response, keratinocyte differentiation, skin microbiome, in vitro atopic dermatitis models, pharmacological therapy

## Abstract

The keratinocyte (KC) is the main functional and structural component of the epidermis, the most external layer of the skin that is highly specialized in defense against external agents, prevention of leakage of body fluids and retention of internal water within the cells. Altered epidermal barrier and aberrant KC differentiation are involved in the pathophysiology of several skin diseases, such as atopic dermatitis (AD). AD is a chronic inflammatory disease characterized by cutaneous and systemic immune dysregulation and skin microbiota dysbiosis. Nevertheless, the pathological mechanisms of this complex disease remain largely unknown. In this review, we summarize current knowledge about the participation of the KC in different aspects of the AD. We provide an overview of the genetic predisposing and environmental factors, inflammatory molecules and signaling pathways of the KC that participate in the physiopathology of the AD. We also analyze the link among the KC, the microbiota and the inflammatory response underlying acute and chronic skin AD lesions.

## 1. Introduction

**Figure 1:**
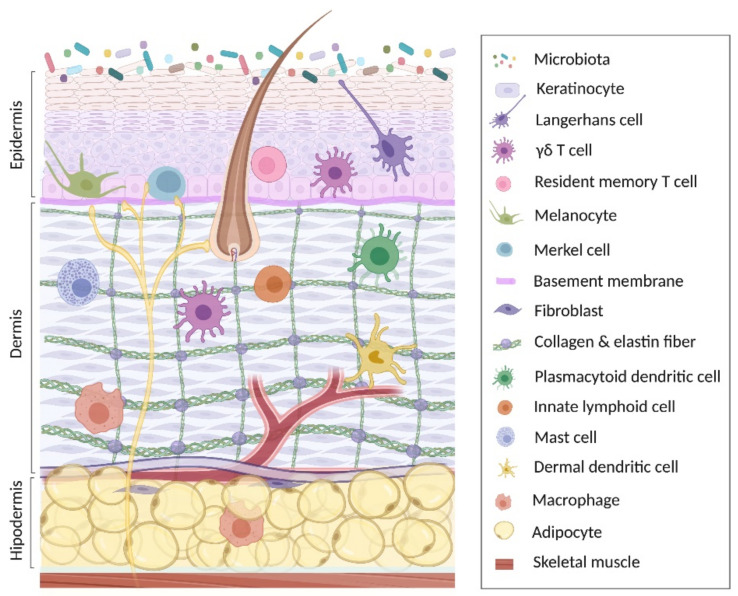
Anatomy of the skin. This image represents the three main layers of the skin, including the most abundant cellular populations in each layer, together with the immune cells present in each anatomical region. Created with BioRender.com (access date: 26 August 2021).

The skin is an organ in constant renewal that covers the body surface. Its total surface area is commonly calculated around 2 m^2^ considering variables such as height and weight; however, folds and invaginations, such as hair follicles and sweat ducts, substantially increase the surface area to 25 m^2^ [1,2]. The skin fulfills specific functions of protection, restraint, thermal regulation and sensitivity, which are carried out in the three layers that constitute the skin (from bottom to top): hypodermis, dermis and epidermis [3] (Figure 1). The hypodermis is the deepest layer of the skin that lies below the dermis and adjoins the deep fascia that covers the skeletal muscle. It is made up of loose connective tissue and stored fat [4], and cells such as fibroblasts, macrophages, and mainly adipocytes. Main functions of the hypodermis are to work as a caloric reserve, thermoregulation and shock absorber [5]. The dermis is located above the hypodermis and is composed of two main extracellular matrix (ECM) components, i.e., collagen and elastin fibers, which are synthesized by fibroblasts. Afferent nerve endings and arteries also penetrate to the dermal deep region. The dermis has notable functions providing tone, strength, resistance, sensitivity and nutrients to the epidermis [6,7]. In addition, this layer of the skin is of great importance since it contains innate immune cells such as dermal and plasmacytoid dendritic cells (DCs), macrophages, mast cells, γδ T cells and innate lymphoid cells (ILC), crucial in the function of defense in both human and mouse [8,9,10,11,12,13]. The epidermis is located more externally and contiguous to the ECM and covers the dermis. It is a semi-permeable stratified keratinized epithelium highly specialized in defense against external agents [3,14]. In addition to keratinocytes (KCs), which are the main functional and structural components of epidermal barrier, Langerhans cells, γδ T cells, resident memory T cells (mainly CD8+), melanocytes and Merkel cells are found in this layer, both in mice and humans [8,10,11,15,16,17,18].

Finally, on the epidermis, the microbiota has an important role in skin health, either modulating the immune response by generating an anti-inflammatory effect or inhibiting the colonization of multiple pathogens [19,20,21]. Skin microbiota is highly diverse and mainly composed by bacterial, fungal, and viral populations, although microbial biomass is relatively reduced compared to that in the gut and airways [22]. Bacterial skin microbiota is mainly represented by three genera: *Staphylococcus*, *Corynebacterium*, and *Propionibacterium*, which represent over 60% of the bacterial species in the skin [23]. Fungal population on the skin is composed by *Malassezia*, *Aspergillus*, *Penicillium*, *Epicoccum*, and *Candida* [24]. Finally, the skin virome has been poorly studied and although respiratory and enteric virus transmission by hand contact is well documented [25,26], viruses are not normal inhabitants of the skin. Studies in healthy skin have demonstrated the presence of cutaneous human β- and γ-papillomaviruses (HPVs), polyomaviruses and circoviruses [27,28]. All these data highlight the great complexity of the skin microbiota.

In the last decade, the KC has become a target of interest in various pathologies, due to its wide versatility. KCs have the capacity of differentiation, regeneration and interaction with various environmental components, as well as with other epithelial components, both structural and immunological, and they function to maintain a state of balance within healthy skin [29,30,31,32]. Alteration of skin homeostasis can generate dermatosis conditions such as psoriasis, seborrheic dermatitis, and atopic dermatitis (AD), among others [33]. All these conditions significantly affect the quality of life of patients. Particularly, AD has aroused particular interest in recent years since records indicate that it affects 15% of the world population. This incidence has been recently altered in the general population, and mainly in health care workers, by the intensive hygiene habits adopted because of the pandemic by SARS-CoV2 [34].

In this review, we summarize the roles of KCs in the context of AD. We first review the biology of the KC, and later review in detail the genetic and immunological profile of the cell that predisposes the beginning and progression of disease, highlighting its interaction with skin microbiota. Current knowledge about the use of the KC as an in vitro model to study AD is also reviewed. Finally, the pharmacological therapies target to restore KC properties in AD treatment is presented.

## 2. Keratinocyte in Skin Homeostasis

**Figure 2:**
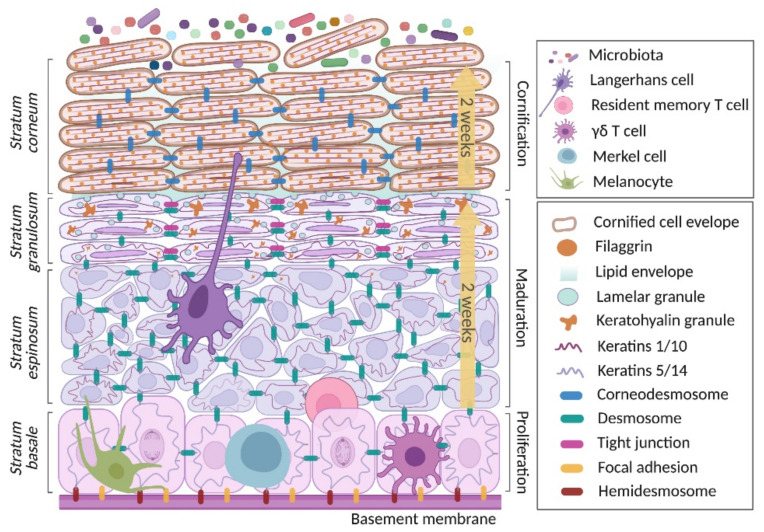
Keratinocyte life cycle. This begins when the keratinocyte (KC) proliferates in the stratum basale. Later, in a course of two weeks, it matures and migrates through the suprabasal layers (spinosum and granulosum stratums) until reaching the top part of the skin, the stratum corneum. Here, the KC acquires the highest degree of maturity and gradually loses viability. Finally, in another two weeks, it moves through the cornified layer to be eliminated by flaking. Created with BioRender.com (access date: 26 August 2021).

The KC is the main component of the epidermal barrier. This cell differentiates as it migrates through the different layers of the epidermis, by extension, from stratum basale (SB), through stratum spinosum (SS) and granulosum (SG) to stratum corneum (SC) [3,35]. During cell migration through these strata, the KC undergoes different processes, including proliferation, maturation (or differentiation) and cornification (Figure 2). In general, the differentiation processes of KCs are widely regulated by calcium, which is related with the fact that the highest calcium concentrations are found in the SB and SC [36,37].

### 2.1. Proliferation Phase

The regeneration of epidermal cells takes place in the epidermal SB, a site where cells are dividing. The basal cells, the main components of this stratum, are cuboidal shaped cells with round to ovoid nuclei and evident tonofilaments within the cytoplasm [38]. In the SB are also located melanocytes that are responsible for skin pigmentation, and Merkel cells that take part in mechanoreception and interact with neurons [15,16,39]. Resident memory T cell and γδ T cells, both with dendritic morphology, are located in this stratum with important implications in local immune responses [40,41].

Basal cells are attached to the ECM of the basement membrane by focal adhesions, mainly β_1_ integrins, and hemidesmosomes (HDs). The latter are composed of the α_6_β_4_ integrin that extracellularly act as a laminin receptor that preferentially binds to laminin-332 [42,43]. α_6_β_4_ integrin is coupled with the transmembrane element bullous pemphigoid antigen (BPAG)-2 (also known as BP180), which act as a functional unity that interacts inside the cell with two plakins, BPAG-1 (also known as BP230) and plectin/HD-1, that form that inner plaque of the HDs and are anchored to cytoskeletal keratin filaments (K)5 and K14 [44,45,46,47]. In turn, keratins modulate the location of the HDs [48]. In vitro, it has been demonstrated that K5 and K14 play important roles in promoting phosphatidylinositol 3-kinase-dependent cell proliferation and negatively modulating Notch1-dependent cell differentiation [49]. To a large extent, the cell migration process begins with the activation of the epidermal growth factor (EGF) receptor (EGFR), since it activates tyrosine kinase Fyn, which phosphorylates the cytoplasmic domain of β_4_, causing the disassembly of HDs [45].

Basal cells undergo asymmetric perpendicular divisions that cause one daughter cell to maintain adhesion with the ECM and the other cell to continue stratification [29], probably in response to biomechanical signaling modulated by cell-cell contact [50,51]. Adherent junctions, which are related to cell-cell adhesion, organization of the cytoskeleton and cell signaling [52], and desmosomes, which are adhesion joints that provide strength to the skin, are also present in this stratum. The former are composed by P-cadherin and E-cadherin, while the latter are constituted by the cadherins desmoglein (DSM) 2 and 3, and desmocollin (DSC) 3 [51,52,53,54].

### 2.2. Maturation Phase

The maturation phase begins when the daughter cells that leave the SB lose the ability to duplicate and begin their migration through the different strata of the epidermis. This process is accompanied by biochemical and morphological changes before cells die in the SC [30,55].

Far from what was previously thought, HDs in conjunction with focal adhesions facilitate KC migration to the SS [56,57]. The SS is characterized by a lower area consisting of several layers of polygonal KCs with a spiny appearance due to the increased amount of newly formed cell-cell junctions, and an upper area consisting of a layer of elongated and flattened KCs parallel to the epidermal surface. Both keratohyalin granules and lamellar bodies begin to appear in the last layer of the SS, although the latter can be only observed by electron microscopy [38,58,59]. Content of both membrane-bound organelles are described later due to their importance in the cornification process. Langerhans cells are also located in the SS and play important functions in the uptake and processing of antigens [11,60]. On the other hand, cells of the SG are elongated and parallel to the surface of the skin, as are their nuclei, with a high content of basic keratohyalin granules. These cells are connected to the SS and SC cells through desmosomes [38]. Tight junctions (TJs), which are structures composed of membrane proteins from the occludin and claudin families associated with plaque proteins such as zonula occludens (ZO)-1 that facilitate the transport of ions and solutes between neighboring cells [61,62], are formed and overexpressed in this layer [61,63]. Living SG cells gradually begin to reduce their viability, and once they reach the SC are known as corneocytes, which are dead cells devoid of nuclei and organelles.

In addition to morphological changes, the differentiation process is accompanied by structural modifications in cell-cell junctions. When KCs exit the basal layer and migrate upwards into the suprabasal layers, they stop expressing K5/K14 to express K1/K10 to form intermediate filaments, which participate in the dynamics of desmosomes and therefore in the stratification of the epidermis, as has been observed in K1 and K10 knockout mice [64]. It has been observed that desmosomes are larger and more abundant in lower layers of the SS than in the upper layers, reducing even more in the SG [65,66]. These variations, as well as the strength of these interactions, are conditioned by modifications in the homophilic (DSC: DSC) or heterophilic (DSC: DSG) expression of proteins, the latter providing greater strength [66,67]. Furthermore, DSG 2 and 3 of the lowest epidermal layers are exchanged for DSG 1 and 4 in the most differentiated layers. On the other hand, DSC 2 and 3 are ubiquitously expressed in the epidermis, while DSC 1 appears from the SS [52]. In relation to TJs, while claudin 1 has been detected in plaques of the epidermis from the SB to SG, with greater intensity in the suprabasal layers, occludin is present in the SG and partly, in the transition cell layer, while ZO-1 is expressed from the SG to the upper layers of the SS [61]. Concerning adherent junctions, P-cadherin is distributed only in the SB, but E-cadherin is evenly distributed in all layers of the epidermis and is essential for TJ formation, but not for desmosome [68,69,70]. On the other hand, gap junction connexin (Cx) proteins are transmembrane channels that communicate adjacent cells [71]. The configuration of the union gap is also related to the differentiation of KCs [72]. Thus, Cx43 is particularly expressed in basal cells, while in more differentiated stages there is a change to Cx26, Cx30 and Cx31 [72,73].

### 2.3. Cornification Phase

Corneocytes are dead cells that present with electrodense, polyhedral, elongated and flattened morphologies, and are joined to each other by corneodesmosomes, serving as the first line of defense against external agents [38,74]. In this layer, the cornified cell envelope (CCE) is formed and reinforced with a lipid envelope. The process of KC migration culminates in desquamation, i.e., the detachment of the corneocytes [30,55].

Cornification is mediated by substances stored in keratohyalin granules and lamellar bodies. The former contain proteins, such loricrin (LOR) and profilaggrin, which together with involucrin (IVL) give rise to the CCE [75,76,77]. Lamellar bodies begin to be released before the establishment of TJs, in the SG [78]. They store lipids, proteins, enzymes and their inhibitors that provide substrates to the SC during the final phase of cornification, and for timely desquamation [79]. They also contain antimicrobial peptides, such as human beta-defensin (hBD) 2, hBD3, cathelicidin LL-37 and ribonuclease 7, that serve as part of the skin microbial barrier [80,81,82]. The corneodesmosomes, junction structures that replace to desmosomes, are widely distributed over the entire surface of the corneocytes of the lower layer of the SC, and to a lesser extent in the superficial corneocytes. These arise when corneodesmosin released from lamellar granules in the SG binds to DSG1 and DSC1, giving rise to the corneodesmosome, which represents an important contribution for the CCE in the replacement of plasma cell membrane with the macromolecular deposition of proteins [76,77]. On the other hand, the profilaggrin released from keratohyalin granules is proteolyzed into filaggrin (FLG) monomers that bind to K1 and K10 filaments of the cytoskeleton, providing mechanical strength and flattering of corneocytes in the most external skin layer [83]. In the intercellular spaces of the SG and SC, large amounts of suprabasin (SBSN), a protein that serves as a substrate for the transglutaminase (TGM) 2 and 3, are deposited [84,85]. By the action of TGM, the corneocytes are then heavily cross-linked and make up the CCE. Later, corneodesmosomes are degraded by kalikrein and other enzymes to initiate natural desquamation, which leads to the establishment of an effective epidermal barrier [76,83,86,87]. Desquamation is essential to maintain the thickness and the self-renewal process of the epidermis [88]. Likewise, in the more superficial cornified layer, nonapoptotic caspase 14 is essential in the catabolism of FLG into hygroscopic amino acids, which are, together with their derivatives, important constituents of so-called natural moisturizing factors (NMF). These FLG degradation products provide moisture, maintain acidic pH and protect from UVB-induced damage [83,89,90].

The SC is elemental in the prevention of transepidermal water loss, a marker of the inside-outside barrier; however, this function is favored by cohesion between the corneocytes and because the corneocytes are covered by a lipid sheet made up mainly of cholesterol, phospholipids, and glycoceramides [91,92]. The production of this lipid envelope has been related to the decrease in pH that characterizes this stratum. A healthy SC has an average pH of 4.7 [93] that is maintained by different mechanisms, among them microbiota colonization [94,95]. Differential distribution of moisture and water retention in the skin favors the establishment of a suitable niche for differential colonization of microorganisms [23].

It has been reported that in the last stages of KC differentiation, and the first stages of cornification, there is an increase in the expression of enzymes and antioxidant elements, which together with the structural proteins are fundamental to the homeostasis of the epidermis [96,97]. The main enzymes detected in the epidermis are superoxide dismutase, glutathione peroxidase, and glutathione reductase; however, lipophilic and hydrophilic nonenzymatic antioxidants, such as α-tocopherol and ubiquinol, or ascorbic acid, uric acid and glutathione, respectively, are also found [98].

## 3. Participation of Keratinocyte in Predisposition, Onset and Progression of the Atopic Dermatitis

AD is a chronic inflammatory disease of the skin characterized by eczematous lesions and a sensation of intense itching. It originates from alteration of the integrity of the epidermal barrier as a consequence of the interaction between genetic and environmental factors [99,100,101,102]. Due to skin barrier defects, there is an increase in the permeability to antigens, both of chemical and protein nature. Penetrating antigens interact with KCs, which are described as hyperactive in people with AD, generating exaggerated responses characterized by an excess of cytokines and chemokines that promotes local inflammatory processes. At the onset, lymphocyte T-helper (Th) response is predominantly via Th2/Th22 with slight Th17 participation [103,104,105], but after chronic allergen exposition, Th1 cells join this enhanced response [104]. The cocktail of cytokines released increases deterioration in the epidermal barrier, inducing disease progression, which is known as the outside-inside-outside hypothesis [106,107,108]. In the following sections, we review the participation on the KC in different stages of AD, including genetic alterations in the cell that predispose to disease onset, and the KC responses to different stimuli that favor the development, maintenance and progression of AD.

### 3.1. Genetic Background of the Keratinocyte Predisposing to Atopic Dermatitis

It is widely accepted that AD is a multifactorial disorder. As aforementioned, the inclination to develop the disease results by the interaction of genetical and environmental factors [109]. Although environmental factors play an important role as triggers of AD, genetic factors stand out for predisposition. Family history strongly impacts on the tendency to develop AD. A study in Hungary demonstrated that 65.5% of children with AD had an ascendency of atopic diseases [110], while in Sweden, parental history of asthma and/or allergic rhinoconjunctivitis and allergy to furred animals and/or pollen increased the odds ratio (OR) to 2.0 for AD in children up to 4 years [111]. Genetic predisposition is a highly determinant factor to increase the susceptibility of developing AD. Genetic alterations related to AD, and in which the KC has important participation, include those that represent epidermal barrier dysfunctions and dysregulation of innate immune response.

The human *FLG* gene (*FLG*) is located in a locus named epidermal-differentiation complex on chromosome 1q21 and, as summarized in Table 1, many polymorphisms and loss-of-function (LOF) mutations in *FLG* generate a high risk of developing AD [102]. In homozygotes or compound heterozygotes LOF mutations of the *FLG* gene, the expressed FLG is impaired, which leads to compromised integrity of the epidermal barrier [83]. Although many mutations have been described worldwide, Asian populations have high frequency of the distribution of these genetic variations on AD patients. Commonly, a LOF mutation in *FLG* acquires more importance in AD onset in the first two years of life, while AD development in childhood or adults may be influenced by other factors [112]. It is surprising that some mutations do not affect the levels of NMF, which may indicate that type 2 skin inflammation causes NMF reduction [113,114].

**Table 1:** Polymorphisms and mutations associated with AD with participation of KCs.

Structural Genes					
Gene	Polymorphism/ Mutations	Mechanism	Prevalent Populations	OR	Reference
*FLG*	P478S	Prevents the protease cleavage through serine phosphorylation, and then affects the FLG aggregation to keratin filaments	Asian (Taiwan) Asian (Korean)	5.67 1.877	[115,116]
	3321delA	Premature termination codon 41 bases downstream that stops protein translation in filaggrin repeat domain 2	Asian	3.54	[112,117]
	S2554X, S2889X, S3296X, K4022X, R501X	Nonsense mutations	Asian	3.54	[112,117]
	2282del4	Deletion of four base pairs that results in a premature stop codon and complete loss of FLG production	Northern European European American African American	NR 5.6 2.5	[118,119]
**Innate immune response**					
*TLR2*	R753Q	Alteration of the function of the intracellular signaling portion homologous to the IL-1 receptor designated as Toll/IL-1 receptor (TIR) domain.	German Italian	NR NR	[120]
	R677W	Associated with reduced NF-κB activation and to increase the risk of bacterial infection			[121]
*TLR4*	D299G	Impaired dimerization of TLR4 and MD-2 in presence of ligand	Italian	2.46	[122,123]
*DEFB1*	A692G	Generates an NF-kB transcription factor-binding sequence in the position -20 of the 5′ untranslated region (5′ UTR) with a plausible effect on the expression of hBD2	Mexican	3.21	[124]
	G1654A	In exon 2, meaning a changed Val37Ile next to six conserved cysteine residues that could affect its folding	Mexican	17.37	[124]
*TSLP*	rs11466749	Means a change 813A/G	European American	0.6	[125,126]
	rs10043985	Means a change 597T/C	African American	0.5	[125,127]
	rs2289276	Means a change 1350C/T	American African	1.8	[125,127]

OR, odds ratio; NR, no reported.

Other genes also expressed in KCs and involved in the innate immune response have been associated with AD development. The Toll-like receptors (TLRs) 2 and 4 have an important role in the activation of the innate immune response on AD [128,129]. For the TLR2 receptor, two clinically relevant single nucleotide polymorphisms (SNPs) with missense changes have been documented, namely R753Q and R677W [130,131], and for TLR4 two SNPs, D299G and T399I, have been described [132]. Another member of the innate immune response secreted by KCs is hBD2 [133]. In the *DEFB1* gene some SNPs have substantial effects on the function of hBD2,with direct impacts on AD onset, namely A692G and G1654A [134,135]. Thymic stromal lymphopoietin (TSLP) is produced by KCs and induces generation of Th2 cells with consequences in the pathogenicity of atopic diseases [136]. Although polymorphisms have been shown to increase the risk for AD (rs2289276), some others have a protective effect (rs11466749 and rs10043985) [125].

Continuous improvement of knowledge concerning polymorphisms that participate in the development of AD, and technological advances in genotyping, will provide better tools to establish strategies for decision making, even before the appearance of the symptoms of AD.

### 3.2. The Keratinocyte in the Primary Origin of Atopic Dermatitis

Some authors indicate that patients in the first stage of the disease present nonlesional skin that is visibly normal and devoid of clinically apparent disease but presents abnormalities in relation to a normal skin [104,137]. Nonlesional AD skin is characterized by increased epidermal thickness and proliferation index, T-cell infiltration, type-2, -22, -17 cytokine and epidermal S100 protein expression, but a reduction in the expression of proteins of KC terminal differentiation, such as LOR, FLG and IVL, as compared to normal skin [104,138]. Anther protein affected in AD development is SBSN, which is also expressed in the epithelial differentiating layers during cornification [84]. Although its function is not fully understood, it is known that shRNA-mediated SBSN knockdown promotes poor formation of keratohyalin granules and affects the development of the SG [85]. In intrinsic AD patients, SBSN is substantially decreased, particularly in those with a nickel allergy [85,139]. These cutaneous abnormalities are exacerbated in lesional skin; thus, nonlesional skin shows an intermediary phenotype between normal and lesional skins, though closer to the latter as immune and epidermal alterations in nonlesional skin are associated with disease extent and severity [104,138]. On the other hand, Th2 cytokines are related to some cutaneous characteristics reported in nonlesional skin of AD patients. Nonlesional skin shows a higher expression of intercellular adhesion molecule-1 (ICAM-1) in basal epidermal cells compared to skin biopsies of healthy individuals. However, when the latter are stimulated with interleukin (IL)-4 there is an increase in expression of ICAM-1 and vascular cell adhesion protein (VCAM), two adhesion molecules which have been associated with the attraction of leukocytes in the lesion area [140]. Gene expression profiling of lesional and nonlesional AD skin has identified signature genes with remarkable roles in physiopathology of AD [141]. An analysis of 127 samples from five different studies demonstrated several differentially expressed genes involved in AD grouped in: (i) epidermal development and barrier function (up-regulated in AD: *KRT16*, *COL6A6*; down-regulated in AD: *LOCE2B*, *LOR*, *FLG*, *SCEL*, *AQP9*); (ii) growth factors and inflammation (chemokines *CCL17*, *CCL18*, *CCL22* are commonly upregulated in AD, while growth factors such as *PGF2*, *EREG*, *OGN*, are down-regulated); (iii) epidermal proteases (*KLK5*, *SERPINB3*, *SERPINB4*, *SERPINB7*, *TMPRSS4*, are up-regulated in AD); (iv) antimicrobial function (*MSMB*, *LTF*, *SCGB2A1* are down-expressed; however, *DEFB4* that codifies β-hBD2 is usually up-regulated likely in response to bacterial infection); and finally, (v) epidermal lipids metabolism (*FADS1*, *FAR2*, *FABP7*, *GPD1* are down-regulated in AD) [142]. Furthermore, human KCs stimulated with IL-4 and IL-13 reduce the expression of the barrier proteins LOR and IVN, that are also diminished in nonlesional skin of patients with AD [143]. However, there is still a lot of controversy about the primary origin of AD, since most of the studies related to this first stage in humans were carried out in areas of nonlesional skin from patients with chronic AD. For this reason, an attempt to explain these mechanisms using human KC cultures or murine models has been made.

**Figure 3:**
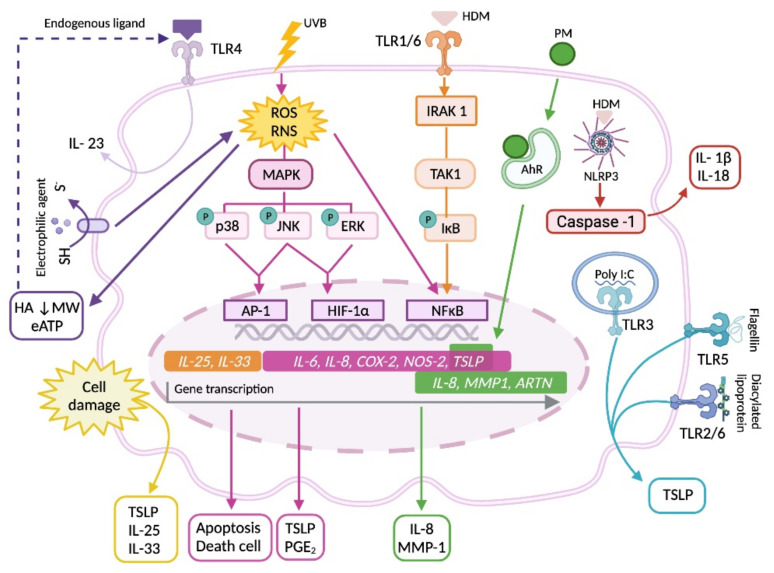
Keratinocyte response to environmental factors. In response to UVB radiation, allergens, endogenous ligands, electrophilic agents, particulate matter present in air pollutants, or ligands of pathogen pattern receptors, KCs activate different signaling pathways that induce apoptosis and cellular death, gene transcription and the release of de novo synthesized type-2 response promoting cytokines and inflammatory mediators, or caspase-1 activation and the subsequent maturation of inflammatory cytokines pro-IL-1β and pro-IL-18. Abbreviations: AhR, aryl hydrocarbon receptor; COX-2, cyclooxygenase-2; eATP, extracellular ATP; HA, hyaluronic acid; HDM, house dust mite; IL, interleukin; MMP-1, matrix metalloproteinase-1; MW, molecular weight; NOS-2, nitric oxide synthase -2; PGE_2_, prostaglandin E_2_; TSLP, thymic stromal lymphopoietin. Created with BioRender.com (access date: 26 August 2021).

In addition to the genetic factors mentioned above, environmental factors such as UVB radiation or pollutants have been associated with the deterioration of the skin (Figure 3). UVB radiation induces the production of reactive oxygen and nitrogen species (ROS and RNS) by KCs, which are involved in apoptosis and cell death, and the synthesis of inflammatory mediators and enzymes, such as IL-6, IL-8, prostaglandin (PG)E_2_ and cyclooxygenase-2 (COX-2), mainly through MAPK, NF-κB and AP-1 pathways [144,145]. Furthermore, studies in other cell populations have shown that ROS also have an important role in the activation of the NLRP3 inflammasome and the subsequent activation of caspase-1 needed for IL-1β secretion [146]. UVB radiation also stimulates TSLP expression in KCs through HIF-1α-dependent mechanisms via the JNK and ERK pathways [147]. Meanwhile, particulate matter (PM) present in air pollutants and mainly composed of a mixture of metals, organic compounds, materials of biologic origin and ions, has been associated with the incidence of AD in young people [148]. Jin et al. demonstrated that PM can enter into human KCs in vitro and induce the synthesis of IL-8 and matrix metalloproteinase (MMP)-1 in a ROS-dependent manner. However, for PM to penetrate the epidermis, barrier-disrupted skin is necessary, as demonstrated in murine models [149]. KCs can be also activated by air pollutants through the aryl hydrocarbon receptor (AhR), contributing to TSLP and artemin expression [150]. On the other hand, antigens or sensitizing agents of an electrophilic nature activate KC signaling through interaction with cysteine residues (thiol groups) of the cell membrane, producing ROS that induce cell death, ATP release and the degradation of skin hyaluronic acid in compounds of low molecular weight that have been reported as endogenous TLR ligands in inflammatory cells [151,152]. As the activation of KCs by endogenous ligands through TLR4 induces the synthesis of IL-23, a cytokine that stimulates the shift from T CD4 lymphocytes to Th22 [153], it might trigger the development of a specific T-response to antigens passing across the deteriorated epidermis and induce AD development. Activation of KCs by house dust mite (HDM) allergens may also contribute to AD onset. *Dermatophagoides pteronyssinus* induces NLRP3 inflammasome activation in a ROS and ATP-independent, but cysteine protease-dependent manner, stimulating KCs to release the proinflammatory cytokines IL-1β and IL-18 [154]. In human KCs and murine models, *Dermatophagoides farinae* extract induces TLR1 and TLR6 activation and promotes the synthesis of the innate proallergic cytokines IL-25 and IL-33 through IL-1 receptor-associated kinase 1 (IRAK 1), transforming growth factor (TGF)-β activated kinase-1 (TAK1), IκB kinase and NF-κB pathways, thus conditioning a Th2 response [155].

**Figure 4:**
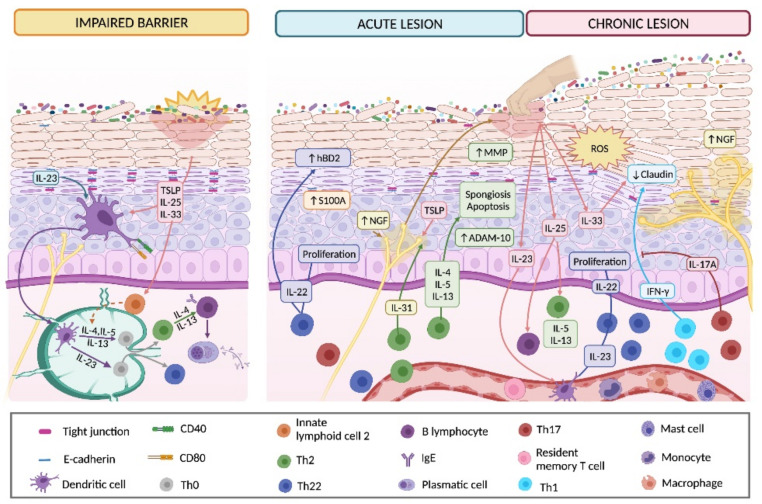
Keratinocyte participation in the onset, development and chronification of atopic dermatitis lesions. Due to an impaired cutaneous barrier, KC-derived cytokines (mainly TSLP, IL-23, IL-25 and IL-33) induce dendritic cell activation and mobilization to nearby lymphatic nodes where they active naïve CD4+ T cell (Th0) and promote Th2 and Th22 polarization. Th2-derived cytokines trigger antibody isotype switching in B lymphocytes to produce IgE. Cytokines produced by Th2 and Th22 cells increase KC proliferation and S100A expression, prompt KC apoptosis and induce cutaneous spongiosis due to a decrease in E-cadherin levels mediated by increased enzymatic activity. These changes are clinically manifested in skin as acute lesions. In this stage, IL-22 and hBD2 mutually enhance their production, perpetuating the inflammatory response associated with AD. Pruritic mediators released by Th2 cells (IL-31) or KCs (NGF and TSLP) increase itching sensation which triggers scratching and worsening of skin lesions. Lesions becomes chronic due to the intensification of the pre-existing Th2 and Th22 inflammatory response enhanced by Th1 and Th17 cytokines. Augmented production of IL-23, IL-25 and IL-33 by KCs maintains Th22 differentiation, up-regulates Th2 cytokine production and diminishes claudin expression, respectively. The decrease in tight junctions, which is enhanced by IFN-γ production, together with the increased levels of NGF, favors epidermal hyperinnervation. Altogether, this inflammatory environment exacerbates the remodeling processes of the epidermis and hyperplasia, while IL-17 down-regulates the IFN-γ effect on claudin expression. Abbreviations: hBD2, human beta-defensin 2; MMP, matrix metalloproteinase; NGF, nerve growth factor. Created with BioRender.com (access date: 26 August 2021).

In murine models, it has been observed that deterioration of the skin by itself induces the production of TSLP [156,157,158], IL-25 [159,160] and IL-33 [161] by KCs. In recent years, TSLP has been proposed as a possible serum marker of AD in humans, together with thymus and activation-regulated chemokine (TARC; chemokine ligand (CCL) 17) [162]. In this context, the production of TSLP can be induced from human KCs by ligands of TLR3 (polyinosinic-polycytidylic acid [poly I: C]), TLR5 (flagellin) or TLR2/6 (diacylated lipoproteins or *Staphylococcus aureus*) [129,163,164], and this effect is enhanced by tumor necrosis factor (TNF)-α, Th2 cytokines and type I interferons (IFNs) [163,165]. The importance of KC-derived cytokines (namely, TSLP, IL-25 and IL-33) is as follows (Figure 4). TSLP actives CD11c DCs, inducing its survival and overexpression of the costimulatory molecules CD40 and CD80 and the chemokines TARC and macrophage derived chemokine (MDC o CCL22) [158,165]. Furthermore, in murine models, TSLP, IL-33 and IL-25 activate ILC2, which is overexpressed in damaged skin [159,160,161,166]. TSLP-activated DCs together with ILC2 induce allogenic naïve CD4+ T cell proliferation and their Th2 polarization to produce IL-4, IL-5, IL-13 and TNF-α [158,160,167], which could trigger atopic march, as shown in murine models [157]. Likewise, in response to IL-4 and IL-13, B cells carry out isotype change of the Ig heavy chain to produce allergen-specific IgE antibodies, which collaborate in allergen up-taken by skin DCs to amplify T cell activation [137].

### 3.3. Involvement of Keratinocytes in Acute Lesions

Acute skin lesions are usually presented as erythematous, itchy papules with serous exudation [168]. As a consequence of continuous scratching, secondary lesions are generated, which include excoriation and crusted erosion. Subacute lesions may also appear as erythematous scaling papules and plaques [169]. These clinical conditions are similar in humans and dogs, thus dogs are used to understand the inflammatory changes underlying AD skin lesions. A transcriptome study carried out in dogs sensitized with HDM showed that, as a result of epicutaneous challenge with an allergen, acute skin lesions were accompanied by a marked genic expression of Th2 and Th22 cytokines, particularly IL-5, IL-13, IL-31 and IL-22, together with the Th2-promoting chemokines CCL5 and CCL17 [170]. In addition to the pruritogenic cytokine IL-31, other genes encoding pruritogenic pathways were also upregulated in dog biopsies, including several proteases (chymase, tryptase, cathepsin S), enzymes involved in leukotriene-synthesis, neuromedin-B, and nerve growth factor (NGF) [170]. This Th2 and Th22 dominance coincides with that manifested in acute AD lesions of patients, which are also characterized by an increase in the levels of expression of a subset of epidermal differentiation complex gene products, mainly S100A7 and S100A8 proteins, in the KCs from the upper SS and SG layers [104]. In humans, robust Th2/Th22 activation is accompanied with some IL-17 skewing, which is significantly higher in children than in adults [105]; however, no up-regulation of Th17 cytokines has been detected in acute AD lesions in dogs [170].

This inflammatory environment influences KC structure and, at the same time, the KC-derived mediators create feedback during inflammation (Figure 4). It has been shown that IL-22 increases the proliferation of KCs and inhibits their differentiation through activation of the MAPK signaling pathway and the decrease in the expression of CCAAT enhancer binding protein-α, a transcription factor that regulates the development, proliferation and differentiation of KCs [171]. Th2 cytokines have a negative effect on the integrity of the KC, since they can induce spongiosis by decreasing E-cadherin expression and increasing the intercellular accumulation of hyaluronic acid and inducing cell apoptosis [108,172,173]. The loss of E-cadherin from the KC surface is generated by an increase in the activity of metalloproteases such as ADAM-10 [173]. Elevated levels of MMP-8 and MMP-9 in the SC from the AD acute lesion, might also contribute to the tissue remodeling process [174]. In the acute inflammatory stage associated with AD, increased serum levels of hBD2 correlated with a high level of IL-22 have been described. Particularly, IL-22 enhances hBD2 production by KCs through activation of the signal transducer and activator of transcription (STAT)-3, and hBD2 increases IL-22 production by CD3/CD28-stimulated T cells via JNK and Akt pathways [174]. hBD2 functions as a chemoattractant of immature DC, memory T cells, macrophages, monocytes and mast cells when binding to CCR6 or CCR2 receptors [175,176,177], with a potential role in the development of the inflammatory and adaptive responses associated with AD. In mice, KC-derived IL-25 induces the release of high levels of IL-13 from ILC2 during AD acute lesions [159]. Finally, in a mice model mimicking human acute AD lesions, the expression of KC-derived IL-33 was augmented and this cytokine was related to an anti-inflammatory effect on the disease [178]; however, in an AD-model in mice overexpressing IL-33, the levels of epidermal claudin 1 were reduced and IL-33 was also able to down-regulate the expression of this TJ protein in KCs assayed in vitro [179], which suggests a possible dual role of IL-33 in AD pathogenesis.

One of the most prominent clinical manifestations of the AD acute phase is pruritus. In this stage, Th2 cytokines, such as IL-4, together with mechanical damage, act synergistically to produce IL-31, a cytokine related to the activation of afferent fibers of neurons in the skin through the IL-31RA receptor [180,181]. In addition, the KC-produced TSLP has the ability to sensitize afferent nerve endings of the skin through the transient receptor potential channels V1 and A1, which trigger a constant itching sensation that induces an increase in the severity of the lesions [182,183]. NGF, produced by KCs and mast cells, is also involved in pruritus, both directly by peripheral nerve sensitization and indirectly by inducing the expression of neuropeptides such as the gene related to calcitonin peptide (cGRP) and substance P [183,184,185,186]. Deeper studies are needed to decipher the exact role of cGRP in AD because recently cGRP has been described as a central negative regulator of ILC2-mediated allergic inflammation [187]. Furthermore, it is known that TJs in the SG are involved in the protection of epidermal nerve endings from external stimuli by nerve pruning, a mechanism that is dysregulated when the epidermal barrier is impaired in an AD lesion, promoting the exposure of nerve endings to itch-inducing agents [188].

### 3.4. Chronic Lesions and KC Participation

If there is a repetitive and persistent exposure to allergens, and the rash and itch of acute lesions progress uncontrolled, patients may develop chronic AD with skin lesions characterized by lichenification and dry fibrotic papules that presents hyperpigmented skin marks [104,168,169]. Even, patients with moderate to severe AD can experience acute and chronic lesions simultaneously. In chronic stages, the inflammatory process becomes difficult to control. An augmentative and vicious circle is generated in which the damaged epidermis and the activated KCs induce skin dysbiosis and generate a cutaneous inflammatory response, accompanied by the sensation of itching, that triggers the desire to scratch, which subsequently enhances barrier disruption and closes the loop [189,190]. At this stage, there is an intensification of the pre-existing inflammatory response. Th2 and Th22 cytokines are over-expressed, although a notable decrease of IL-4 and IL-4R is detected, accompanied by an upregulation of markers of the Th1 response, mainly IFN-γ and, in a lower degree, of Th17 [104,137,191,192]. In this regard, it has recently been shown that the IL-4α receptor blockade upregulates IFN-γ-producing cells after activation of lymphocytes from AD patients with staphylococcal enterotoxins B [193].

As shown in Figure 4, the large amounts of IL-25 released by KCs in chronic lesions maintain the production of IL-13, mainly by skin accumulated CD4+ T cells, which contributes to the remodeling processes of the epidermis and hyperplasia development [159]. Mediators released from Th22 cells have also been associated with skin remodeling mechanisms by in vitro assays [194]. The constant lesions generated by scratching induce the production of IL-23 by KCs, which have been shown to induce endogenous expression of IL-23 by DCs that, in turn, shift naive CD4+ T cells to Th22 differentiation, which releases high levels of IL-22 causing hyperplasia of the epidermis and chronicity [153,171]. In addition, epidermal hyperinnervation, which is thought to underlie pruritus, has been observed in patients with AD. Analysis of skin biopsies from patients with chronic AD suggests that this may be due to an increase in NGF production and a decrease in the expression of the epidermal innervation regulatory protein semaphorin A3 [195]. Thus, experimental models of dry-skin, both in vivo and in vitro, have demonstrated that a decrease of epidermal nerve densities generates antipruritic effects [196]. In relation to Th1 and Th17 cytokines, little information exists about their effects on KCs in chronic AD. Recently, IFN-γ has been shown to inhibit claudin 1 expression via the JAK/STAT signaling pathway in normal human KCs, which is reflected in the loss of TJ function in a model equivalent to human skin [191]. Strikingly, IL-17A, which is also abundant in chronic lesions of AD, is able to revert TJ dysfunction induced by IFN-γ [191]. On the other hand, it has been observed that after KC damage, large amounts of ROS are expressed in skin biopsies, intensifying inflammatory responses and aggravating skin pathologies such as AD [197]. In summary, all these mechanical and immunological stimuli cause hyperplasia of the epidermis and prominent hyperkeratosis with minimal spongiosis, shaping the characteristic lesions of chronic AD [198]. However, more studies are needed to understand the predominant cellular and molecular mechanisms underlying AD chronic lesions, which will allow a better clinical follow-up of the patient and more efficient treatments.

### 3.5. Bidirectional Communication between Keratinocyte and Microbiota

Intercommunication of the KC with the complex microbiota that inhabit the skin is a relevant factor in maintaining the balance between health and disease. More than a commensal relation, microbiota and KCs work in a dynamic manner to generate a favorable ecosystem for both parties. However, when this relationship is interrupted, the normal functions of the skin are altered, making it prone to developing skin diseases.

**Figure 5:**
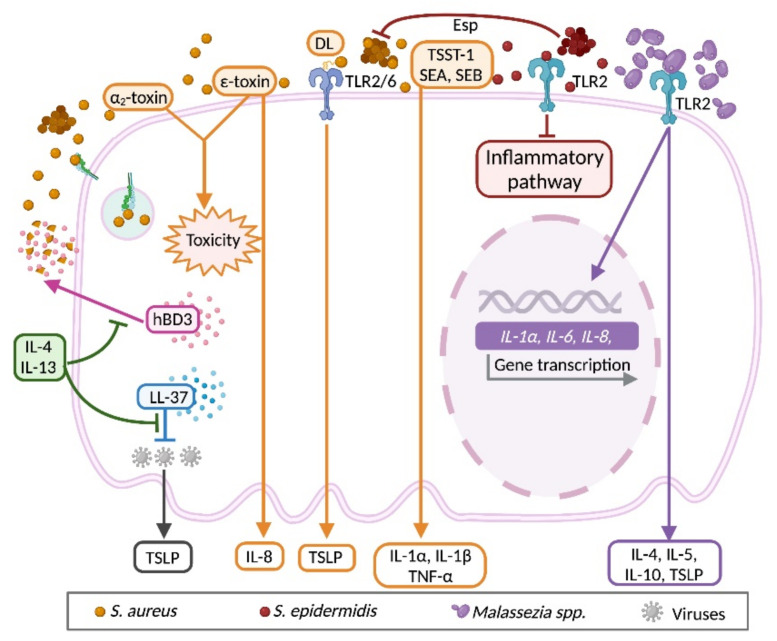
Keratinocyte response to microbiota stimuli under the type 2 immune milieu. KCs recognize bacteria, fungi, and viruses from skin microbiota through specific receptors. Colonization with *Staphylococcus aureus* is present in the skin of AD patients, and the bacteria or their components can activate KCs to secrete proallergic (TSLP) and proinflammatory (IL-1α, IL-1β, IL-8, TNF-α) cytokines, exacerbating the underlying immune response. Exotoxins from *S. aureus* can also damage the cell. Chronic infection of *S. aureus* is partly due to its internalization into KCs through extracellular adherence protein recognition. Th2 cytokines (IL-4, IL-13) avoid *S. aureus* destruction by KC-derived hBD3. As *Staphylococcus* *epidermidis*/*S. aureus* ratio is diminished in AD skin, the anti-inflammatory and protective effects, and the anti-microbial properties of *S. epidermidis* on KCs and *S. aureus*, respectively, are impaired. In response to *Malassezia* colonization, KCs express proinflammatory IL-1α, IL-6, and IL-8, and release type 2 (IL-4, IL-5, IL-10, TSLP) cytokines. Levels of LL-37 in KCs are down-regulated by type 2 cytokines, increasing viral replication and dissemination of vaccinia, herpes simplex and human papilloma viruses, which at the same time promotes type 2 inflammation through an induction of TSLP expression by the cell. Abbreviations: DL, diacylated lipopeptide; Esp, serine protease; hBD3, human β-defensin 3; LL-37, cathelicidin LL-37; SEA, staphylococcal enterotoxin A; SEB, staphylococcal enterotoxin B; TNF, tumoral necrosis factor; TSST-1, toxic shock syndrome toxin. Created with BioRender.com (access date: 30 August 2021).

Members of the genus *Staphylococcus* are common inhabitants of the skin; however, staphylococcal infections are frequent in impaired skin in AD. Colonization with *S. aureus* is present in 70% of AD patients with skin lesions and 39% in those with nonlesional skin [199]. *S. aureus* exacerbates the inflammatory process in AD patients by direct interaction of its structural components and released exotoxins with molecular receptors of KCs (Figure 5) [200]. In human primary KCs, TLR2/TLR6 heterodimer activation by diacylated lipopeptides of *S. aureus* induces mRNA expression and release of TSLP [129]. Moreover, Th2 cytokines IL-4 and IL-13 inhibit KC mobilization of hBD3 from the cytoplasm onto the bacterial surface of *S. aureus* [201], which, in turn, favors the continuous activation of TLR2/TLR6 and, consequently, sustained production of TSLP and more Th2 immune milieu. On the other hand, *S. aureus* produces and secretes toxins (exotoxins) that damage target cells (cytotoxins) or induce exacerbated cytokine production by stimulation of T cell (superantigens) [128]. For example, secreted α2-toxin, also known as phenol-soluble modulin-α2, triggers a potent induction of cell toxicity in KCs isolated from mice [202,203]. In addition, staphylococcal ε-toxin is cytotoxic to KC and causes a proinflammatory reaction by induction of IL-8 by the cell, as well as delaying the proliferative capacity of immortalized human KCs [203]. Superantigens have also been found to activate KCs. In cultured KCs from lesional skin of AD patients, increased expression of IL-1α, IL-1β and TNF-β is induced after staphylococcal enterotoxins A or B, and toxic shock syndrome toxin-1 stimuli, as compared to those from nonlesional skin or from normal skin of nonatopic patients [204]. Altered cytokine secretion in superantigens-challenged KCs may explain the increased inflammation in staphylococcal lesional skin. It is worthy of note that intracellular *S. aureus* invasion is a critical factor that promotes the persistence of chronic infections, since it allows evasion to antibiotic treatments [205]. Bacterial adhesins in *S. aureus* allow bacterial adhesion to KCs and eventually internalization. Exposure of primary KCs to the secreted extracellular adherence protein from *S. aureus* favors bacterial adhesion and internalization, a mechanism that might be mediated by fibronectin as a bridging factor, and integrin α5β1 in KCs [206].

*Staphylococcus epidermidis*, a member of the coagulase-negative staphylococci, together with the species *Staphylococcus hominis*, *Staphylococcus haemolyticus*, *Staphylococcus capitis*, *Staphylococcus lugdunensis* and *Staphylococcus warneri*, is one of the most prevalent components of the skin microbiota ubiquitously distributed [207,208,209]. Although *S. epidermidis* is considered a commensal of the skin, eventually it may act as an opportunistic pathogen [210,211,212], and can even act in a mutualist manner with KCs [94]. Once *S. epidermidis* has colonized external KCs, they stablish a commensal habitat by competing for substrate with other potential pathogens, such as *S. aureus*. Direct production of antimicrobial metabolites has been reported in *S. epidermidis*. The so-called inhibitory-type *S. epidermidis*, isolated from half of volunteer nasal cavities, can reduce biofilm formation of *S. aureus*. This inhibitory capacity is attributed to the* S. epidermidis* serine protease (Esp), which possess serine-protease activity. Remarkably, the microbicidal activity is most observed when combined with hBD2 secreted by KCs [133]. In an experimental model of nasal infection with methicillin-resistant S. aureus (MRSA), preinoculated mice with Esp-secreting *S. epidermidis* prevented the nasal colonization by MRSA [213]. In addition to interfering with the colonization of other bacteria on the KC, *S. epidermidis* can modulate the inflammatory response of the KC. It has been demonstrated that the stimulation of primary human KCs by poly I:C through the TLR3 receptor induces the release of inflammatory cytokines such as TNF-α. Noteworthy is that poly(I:C)-treated KCs reduce the production of TNF-α when exocellular lipoteichoic acid from *S. epidermidis* (LTA-Se) is added. Moreover, in vivo experiments have demonstrated that LTA-Se prevents wound-induced IL-6, TNF-α and inflammation in wild-type mice, but this effect is missed in Tlr2−/− mice, suggesting that TLR2 stimulation by LTA-Se prevents inflammatory responses in KCs [21]. Another important relation between KCs and *S. epidermidis* is the capacity to provide an adequate substrate and provide metabolites that improve protection. Autologous application of *S. epidermidis* on the skin of healthy subjects helped to improve skin moisture retention and to keep low acidic skin conditions through the secretion of glycerin, and lactic and propionic acids [94]. These results open the possibility of evaluating *S. epidermidis* application as a new therapy in AD patients.

The main representative of the fungal flora of the skin is lipophilic yeast of the genus *Malassezia* spp. [214]. However, *Malassezia* spp. can behave as opportunistic pathogens and are commonly presented in skin lesions of adult AD patients [215]. Of the nine species found in healthy patients, four of them are frequently presented in patients with mild and severe AD, such as *Malassezia globosa*, *Malassezia restricta*, *Malassezia sympodialis*, and *Malassezia furfur* [216,217,218]. Skin inflammatory processes in AD can be also mediated by the interaction of KCs with *Malassezia* species due to these fungi modulating the production of proinflammatory mediators [219]. In vitro interaction of the human KC cell line HaCaT, (human, adult, low calcium, high temperature) with *Malassezia* species (*M. furfur*, *M. globosa* and *M. restricta*) induces the expression of IL-8, IL-6 and IL-1α, and this effect is mediated by the activation of TLR2 [220]. Some *Malassezia* species are responsible for the production of type 2 cytokines in KCs. The human primary KCs exposed to *M. globosa* result in an increment of IL-5, IL-10 and IL-13 secretion, whereas *M. restricta* induces IL-4 secretion [221]. As previously mentioned, TSLP has an important role on the induction of the type 2 inflammatory response, and its production has been demonstrated to be induced by *M. globosa* and *M. restricta* in KCs, which might be mediated by the activation of lysophosphatidic acid receptors 1 and 3 of KCs by the lipid layer of *Malassezia* [222].

The impaired skin barrier and the type 2 inflammatory response are important factors that allow higher susceptibility to viral infection in patients with AD [223]. For example, skin from AD patients is characterized by overexpression of the type 2 cytokines IL-4 and IL-13 and, conversely, production of type 1 cytokines IL-12 and IFN-γ is reduced, which in turns reduce the expression of the human cathelicidin LL-37 [224]. This condition makes KCs highly susceptible to experimental infection with the vaccinia virus, which is why AD patients are commonly excluded from smallpox vaccinations since the risk developing eczema vaccinatum is significantly increased [225,226]. Moreover, eczema herpeticum results from the dissemination of herpes simplex virus (HSV)-1 or -2 in AD patients [227]. In vitro studies have demonstrated that, in the presence of LL-37, the replication of HSV-2 in primary human KCs is reduced in a dose-dependent manner [228]. An altered antiviral innate immune response in KCs has been proven to be due to reduced levels of the transcription factor specificity protein 1, which is related to an enhanced replication of vaccinia virus and HSV in AD patients and leads to the overexpression of TSLP and six members of the family of human kallikreins in the KCs [229,230]. The frequency of AD is also potentially related to the susceptibility and persistence of high-risk HPVs [231]. Certainly, compromised epithelial barrier function in AD makes an easy target for viral infection, and some viruses express proteins able to induce changes toward atopy. In transgenic mice (K14.E7), the expression of the oncoprotein E7 from HPV16 driven by the K14 promoter, which restricts the E7 expression to epithelial cells in the skin, results in the characteristic lesions of AD accompanied by increased production of TSLP and total IgE [232]. Viral dsRNA and a Th2 cytokine milieu might promote type 2 inflammation through an induction of TSLP expression by KCs, suggesting the existence of a vicious cycle between AD and viral infections [163]. In the case of the measles virus, some studies have described an increase in the incidence of AD after vaccination [233], while others have shown a decrease in the risk of AD after measles infection [234], or an improvement in skin lesion or immunological parameters after natural infection or vaccination [235,236]. This controversy was addressed considering the role of KCs in the induction of atopy. When HaCaT cells were exposed to the measles virus, the expression of TSLP and CCL26 (eotaxin-3, a chemotactic factor for eosinophils and basophils) was diminished, while TGF-β was overexpressed. In the same report, a clinical protocol in which patients with moderate AD received a measles vaccination demonstrated that lesional skin reduced the expression of TSLP and CCL26, which was accompanied with reduction of clinical scores of AD [237,238].

Although growing evidence about the interaction of skin with microbiota is more available every day, complete understanding of this complex relationship remains one of the major goals in the field. Undoubtedly, the discover of all mechanism generated by KC-microbiota interactions will provide insight into immunomodulatory activity in AD.

## 4. The Use of the Keratinocyte as an In Vitro Model of Atopic Dermatitis

The great versatility of KCs has allowed their use as a model to study multiple pathological processes, including AD. These models are highly variable, but can be grouped into two- or three-dimensional groups.

Two-dimensional monolayer cultures are based on the adherence of cells to a glass or polystyrene surface that provides a mechanical support for the cells [238]. In this way, the response of KCs to any damage can be specifically and easily evaluated. The sources of these cells are usually primary human KCs from a healthy patient or with AD, or immortalized human KCs [239]. Monolayer cultures of KCs are achieved due to epidermal differentiation of KCs as a consequence of confluence, cell-cell interactions, or specific factors such as calcium or EGF under conditions of subconfluence [114,240,241].

The advantage of working with KCs from patients with AD as in vitro model of the pathology, is that these cells are already genetically modified and conditioned for the development of pathology [239]. However, the pathological status of AD can be mimicked using normal human epidermal KCs (NHK) or immortalized cell lines. The most widely used cell line for this type of model is HaCaT cell line, that harbors nononcogenic mutations in the *TP53* gene [242], and retains its differentiation properties [243]. It is known that the exposure of KCs to type 2 cytokines, mainly IL-4 and IL-13, induces their differentiation to the pathophysiology conditions of AD. Accordingly, HaCaT cells exposed to both IL-4 or IL-13 attenuate the expression of genes that regulate epidermal cell structure and barrier function in the terminal stage of KC differentiation, such as proteins highly expressed in the SS and SG of healthy skin: K1, K10, DSM 1 and DSC 1. The effect is mediated through mechanisms dependent on the receptor IL-4Rα and STAT-6 at early stages of KC differentiation [244]. Furthermore, human primary KCs treated with both IL-4 and IL-13 reduce the gene and protein expression of FLG; contrary to up-regulated expression generated by IFN-γ exposure [114]. Likewise, Dang et al. demonstrated that the silencing of *FLG* in NHKs in vitro causes a reduction in the protein expression of K5, K10 and K14, IVL, and TGM 1, along with an increase in LOR, altering the function of the cutaneous barrier mainly at the SC layer level [245]. Furthermore, *FLG* silencing was accompanied by an increase in the generation of IL-4, IL-5, IL-13 and IL-2, as well as a decrease in IL-12 and IFN-γ. In response to cytokines, NHK and HaCaT cells modify in a different manner the gene expression of proteins associated with the CCE. Thus, NHK cells stimulated by IFN-γ, IL-4 or IL-17A, or to a lesser extent with IL-22, decrease the expression of FLG, LOR and K10, and only when stimulated by IFN-γ a significant increase in TGM 1 and TGM 2 is detected [246]. HaCaT cells diminish the gene expression of *K10* and *IVL* only in response to IL-4, and show an increase in *FLG* and *TGM 2* [247], suggesting that HaCaT cells might be a poor model to study the integrity of the epidermal barrier under a cytokine-dependent environment. However, hBD2 transcriptional profiles in response to IFN-γ, IL-17A or IL-4 were similar between HaCaT cells and NHKs, being up-regulated in response to the first two cytokines and down-regulated upon stimulation with IL-4 [246,247].

As previously mentioned, various in vivo experimental studies have shown that AD is a very complex pathology. Although it is characterized by the prevalence of a Th2 environment, IFN-γ has been identified as a crucial cytokine in AD patients with chronic lesions. Therefore, it has been proposed that several in vitro models based on KC stimulation with cytokines can be used for a greater approximation to what really happens at molecular level in AD. Thus, the stimulation of HaCaT cells with IFN-γ together with TNF-α for 6 h induces the expression of IL-1β, IL-6, CCL17, CCL22 and IL-33 at the gene and protein level [248]. Furthermore, after 24 h of exposure with the same cytokines, the up-regulated expression of CCL17, CCL22 and IL-33 is maintained, and a reduction in *FLG* and *LOR* mRNA levels is observed. When cells are stimulated with IL-4, only an increase in IL-13, IL-5 and IL-25 is shown, a phenomenon that is reversed when IL-4 is combined with TNF-α [249]. Therefore, as long as the characteristics of the problem are adequately defined, the stimulation of cultured HaCaT cells with cytokines is a good approach for studying the cellular and molecular aspects of AD. Thus, as the experimental conditions get closer to the real pathophysiology, a better understanding of the disease will be obtained. However, the monolayer models present various limitations, prime among which is their lack of stratification; therefore, the interest in the development and characterization of the three-dimensional models is growing.

Three-dimensional models of human reconstructed epidermis arise from the differentiation of normal human KCs in a chemically defined medium under exposure to an air-liquid interface [250]. There is a variant where the differentiation of KCs starts in a matrix formed by fibroblasts and collagen [251]. Both models emerge as possible strategies in the study of pharmacological treatments for lesions of various origins. Particularly, addition of inflammatory molecules enables the creation of a compromised RE model presenting many AD-like characteristics, such as abnormal differentiation, higher secretion of proinflammatory molecules by KCs and a specific gene expression pattern [252]. In this context, both the IL-4, IL-13, IL-31, TNF-α and the IL-4, IL-13, poli I:C, TNF-α inflammatory cocktails induce edema, better known as spongiosis, within the lower layer of the epidermis of a human reconstructed epidermis model, as well as TSLP secretion by KCs and the down-regulation of *FLG* gene expression [252,253]. In relation to lipid composition, TNF-α alone or combined with Th2 cytokines and IL-31 only affects SC lipid composition, mimicking changes observed in AD patients [253].

## 5. Pharmacological Therapy to Restore Keratinocytes

The use of topical agents is still the strategy of choice for the treatment of AD. Although severe cases require a combinatory regimen with phototherapy, systemic antibiotics, immunomodulatory drugs or monoclonal antibodies, the modulation of the KC response in AD is key for patient recovery. Mostly, systemic therapies are directed to achieve a systemic immunomodulation of the cellular and molecular elements involved in the type 2 response [254]. However, the KC is a cell that may actively induce exacerbation of the AD symptoms by the production of proinflammatory and prurigenic/pruritic mediators, so many of the topical treatments are focused on reducing this response.

Moisturizer products are highly recommended to improve moisture content in the SC, which is decreased in AD, as they help to treat xerosis and prevent allergen invasion and relapse of dermatitis, as well as suppression of itching by recovering and maintaining skin barrier functions [255,256]. In a randomized controlled trial of infants with moderate to severe AD, it was revealed that regular emollient use reduced the need for topical corticosteroid use and improved symptoms [257]. The use of topical corticosteroids together with emollients is a valuable therapeutic approach to reduce inflammation and pruritus. Hydrocortisone increases the activity of fatty acid synthase, a key enzyme in fatty acids synthesis in KCs in the SG that promotes the secretion of free fatty acids [258]. Topical betamethasone was shown to normalize epidermal differentiation and reduce epidermal hyperproliferation, although it led to epidermal thinning [259]. Betamethasone also promoted a diminution of the transepidermal water loss in AD patients [259]. However, in a post-treatment phase, an impairment of skin barrier function was measured as the rates of water accumulation returned to initial levels [260]. Although these results indicate the effect of betamethasone on skin hydration, it might also modify the induction of the type 2 cutaneous response since it is able to downregulate TSLP expression in NHK [261]. Although antihistamines are not recommended, they still represent the therapeutic regimen of choice among dermatologist for the treatment of pruritus [262]. Particularly, sedating antihistamines are used in the pediatric population to help patients with a negative impact of AD on sleep. Cetirizine, a histamine H1-receptor antagonist, has shown an anti-inflammatory effect in vitro that is mediated by the reduction of IL-8 production on the macrophage migration inhibitory factor (MIF)-stimulated human KC A431 cell line. Moreover, in a direct form, cetirizine also inhibits MIF production in A431 cells [263]. However, no evidence of improvement of clinical signs of AD was observed in patients treated with oral H1 antihistamines, included cetirizine, as an adjuvant therapy alongside topical agents [264].

Although topical corticosteroids are the standard treatment in AD, inhibitors of calcineurin topically applied, mainly tacrolimus and pimecrolimus, are broadly used since few adverse effects have been reported [265]. Although tacrolimus (FK506) has a relatively high molecular weight (822 Da) and shows good skin penetration activity, it is commonly used topically in conjunction with paraffin-based ointments [266]. The immunomodulatory effect of tacrolimus is mediated after binding immunophilins (mainly FK506-binding protein-12, FKBP-12) and the formed complex (tacrolimus – FKBP-12) then binds to the phosphatase calcineurin and inhibits phosphatase activity, which in turn prevents the nuclear factor of activated T cells (NFAT) dephosphorylation and translocation to the nucleus [267,268,269]. Hence, it suppresses the activation of T cells by reducing the production of IL-2 and other proinflammatory cytokines. The anti-inflammatory activity of tacrolimus has been observed in KCs. When NHKs are exposed to UVB, the secretion of TNF-α is significantly increased, as in the milieu of AD [270,271]. However, when KCs are treated with tacrolimus, the activation and translocation of NF-kB is reduced in a dose-dependent manner that downregulates the production of TNF-α [271]. Moreover, in TNF-α-stimulated NHKs, the secretion of TGF-β is incremented by tacrolimus and, conversely, the expression of inducible nitric oxide synthase (iNOS) is downregulated, which probably is associated with its therapeutic efficacy in the treatment of AD [272]. The chemokine RANTES (regulated on activation, normal T expressed and secreted) is a potent inductor for eosinophils that is increased in lesional skin of AD patients and is produced by KCs after stimulation with inflammatory cytokines [273]. In Korean AD patients, daily treatment with 0.03% tacrolimus ointment for 8 weeks significantly reduced the number of RANTES-positive cells in lesional skin [274]. In human KCs, the overexpression of RANTES induced by IFN-γ and IL-4 is significantly reduced by 10^−8^ or 10^−6^ M of tacrolimus, indicating its possible role in the amelioration of AD through KCs targeting [275]. On the other hand, the pimecrolimus (DZ ASM 981) immunomodulatory mechanism of action is similar to that of tacrolimus [267], although less information is available concerning its effects on KCs. In a model of 2,4-dinitrochlorobenzene (DNCB)-induced AD in NC/Nga mice, topical treatment with pimecrolimus reduced the expression of TSLP [276], which, as previously mentioned, is an IL-7-like cytokine highly expressed in KCs after stimuli [277]. In addition, pimecrolimus impaired the activation of NFAT2 in human KCs from the outer root sheath and some of the inner root sheath of the hair follicles [278].

Finally, phosphodiesterase 4 (PDE4) is an enzyme degrading cyclic adenosine monophosphate (cAMP). Among PDE4 inhibitors, apremilast and crisaborole have been broadly used in the treatment of AD, proving to be modest to highly effective in cases of moderate to severe AD [279,280,281]. In AD patients, PDE4 is highly active in mononuclear leukocytes [282]. Moreover, in IL-1α-stimulated primary human KCs, the mRNA expression of IL-8 and TNF-α is reduced in a dose-dependent fashion in presence of the novel and selective PDE4 inhibitor DRM02 [283]. However, posterior evidence has demonstrated that mRNA expression of TNF-α, IL-1α and CXCL8 in PGE_2_-treated NHKs treated with apremilast is reduced, without changes in phosphorylation of the cAMP-PKA-CREB pathway, suggesting an alternative cAMP-independent mechanism that down-regulates these mediators [284]. In human KCs, apremilast reduces the expression of the inflammatory mediators IL-12/IL-23p40, IL-31, CCL5, and alarmins S100A7, S100A8 and S100A12, under stimulation of the type 2 cytokine IL-4 [285]. Then, PDE4 inhibitors demonstrate favorable improvement on the severity of AD by targeting the response of KCs.

## 6. Conclusions

AD is a heterogeneous skin disease characterized by skin barrier dysfunction, skin inflammation and intense pruritus. Although great advances in the understanding of this cutaneous disease have been achieved over the last years, its pathogenesis is still enigmatic, resulting in a lack of specific treatments. This is further complicated by the lack of data to address whether acute and chronic AD represent progressive stages across a continuum of inflammatory responses, or if each has distinct immunologic mechanisms and diversity among individual patients. However, it is evident that the complex interplay of KCs with environmental agents, skin microbiota, inflammatory cells, and nerves is critical in AD onset, development, progression and chronicity. Current advances in understanding the accurate participation of KCs in AD pathogenic mechanisms may facilitate new drug development, as KC restoration improves local immune dysregulation and avoids cutaneous infection.

## Data Availability

Not applicable.
